# Factors associated with medical students’ scores on the National Licensing Exam in Peru: a systematic review

**DOI:** 10.3352/jeehp.2022.19.38

**Published:** 2022-12-29

**Authors:** Javier Alejandro Flores-Cohaila

**Affiliations:** 1 Academic Department, USAMEDIC, Lince, Peru; 2 Peruvian University Cayetano Heredia, San Martín de Porres, Peru; 3 Red EsSalud Tacna, Tacna, Peru; Hallym University, Korea

**Keywords:** Medical students, Medical education, Peru, Educational measurement

## Abstract

**Purpose:**

This study aimed to identify factors that have been studied for their associations with National Licensing Examination (ENAM) scores in Peru.

**Methods:**

A search was conducted of literature databases and registers, including EMBASE, SciELO, Web of Science, MEDLINE, Peru’s National Register of Research Work, and Google Scholar. The following key terms were used: “ENAM” and “associated factors.” Studies in English and Spanish were included. The quality of the included studies was evaluated using the Medical Education Research Study Quality Instrument (MERSQI).

**Results:**

In total, 38,500 participants were enrolled in 12 studies. Most (11/12) studies were cross-sectional, except for one case-control study. Three studies were published in peer-reviewed journals. The mean MERSQI was 10.33. A better performance on the ENAM was associated with a higher-grade point average (GPA) (n=8), internship setting in EsSalud (n=4), and regular academic status (n=3). Other factors showed associations in various studies, such as medical school, internship setting, age, gender, socioeconomic status, simulations test, study resources, preparation time, learning styles, study techniques, test-anxiety, and self-regulated learning strategies.

**Conclusion:**

The ENAM is a multifactorial phenomenon; our model gives students a locus of control on what they can do to improve their score (i.e., implement self-regulated learning strategies) and faculty, health policymakers, and managers a framework to improve the ENAM score (i.e., design remediation programs to improve GPA and integrate anxiety-management courses into the curriculum).

## Introduction

### Background

Licensing examinations in medicine are widespread across the world [1]. These examinations let us know that our doctors have achieved a minimum of knowledge and skills to provide quality care [2]. In addition, a higher score on a licensing examination may be associated with better access to residency programs, better work opportunities, and patient safety [3].

For Peruvian medical students, the licensing examination is called the National Licensing Exam (ENAM) [4]. Developed in 2003 by the Peruvian Society of Medical Schools, it remains the standard to ensure that doctors can practice medicine in Peru. The ENAM is a multiple-choice question exam, with a total of 180 questions mostly in the form of clinical cases based on the most important disease in Peru. Due to regulatory mechanisms and to increase its importance, the ENAM is now the biggest contributor to the selection of the Rural Service in Peru, and it has an influence on the selection of medical specialties in Peru [5,6].

In addition to its regulatory role, the ENAM informs the population on the level of knowledge of our future doctors. However, in 2021, a study conducted by Mendoza et al. alarmed medical educators, health policymakers, and the general population, mainly because they found a high rate (42.8%) of disapproval [7]. Although it was an important study, there was little medical education theory involved in the design and interpretation of results.

It is widely known that medical education research must be strongly based on theory, models, or a framework at the moment of design or analysis [8]. For early-career medical education researchers, theory may appear difficult and disturbing [9]; this phenomenon is well represented in the ENAM-related research and has led us to poor understanding of which factors influence ENAM scores.

### Objectives

Therefore, this review aimed to identify which associated factors have been studied with respect to ENAM scores in Peru, and to develop a framework to explain how these factors interact with the final score.

## Methods

### Ethics statement

As this study was a systematic review, no human or human-origin materials were involved; thus, neither approval by the institutional review board nor the obtainment of informed consent is required.

### Study design

This systematic review was described according to the Preferred Reporting Items for Systematic Reviews and Meta-Analyses (PRISMA) statement, available from: http://www.prisma-statement.org [10].

### Eligibility criteria

The inclusion criteria for this systematic review were: (1) studies published in English or Spanish; (2) studies published since 2003; (3) quantitative studies (analytical cross-sectional, case-control, cohorts, quasi-experimental, and experimental studies); and (4) studies that assessed the ENAM score as an outcome. Qualitative studies, and not fully accessible studies were excluded.

### Information sources

The search was conducted in September 2022 in 4 databases: MEDLINE, EMBASE, SciELO, and Web of Science. One register was searched: the National Register of Peruvian Research (RENATI, abbreviation in Spanish). To assess the gray literature, the first 10 pages of Google Scholar were assessed. Finally, all references of eligible studies were analyzed to identify further studies.

### Search strategy

The search strategy was developed in PubMed (MEDLINE) and translated to other databases using a polyglot tool (https://sraccelerator.com/#/polyglot). The main search was:

#1: ENAM: ENAM or “Licensing Examination” OR “National Licensing Exam”

#2: Associated factors: correlation OR concordance OR differences OR association OR “associated factors”

#3: Peru: Peru OR Peruvian

#4: #1 AND #2 AND #3

For SciELO, Web of Science, RENATI and Google Scholar, only part #1 of the string was applied (Supplement 1).

### Selection process

The author (J.F.C.) eliminated duplicates using Zotero ver. 6.0 (Digital Scholar, Vienna, VA, USA). The remaining studies were assessed using the eligibility criteria to check the titles and abstracts. The full texts of eligible studies were evaluated, and the reference lists of these articles were reviewed to identify further studies.

### Data collection process

The main author (J.F.C.) collected the data from selected studies using an extraction form prepared that included general data from studies, specific data using the academic achievement model by Alyahyan and Dustegor [11], and inferential data from studies.

### Data items

As specified before, the data extracted were as follows: (1) general data from studies: first author, year, city of provenance, study objective, type of study (journal article, thesis, or other) and study design; (2) specific data: sample size, students’ sociodemographic characteristics (age, gender, nationality, marital status, and academic status), socioeconomic status, students’ environment (medical school, internship setting), learning activities (study resources, preparation time, others), psychological factors, measures of prior academic achievement (grade point average [GPA], other) and ENAM score; and (3) inferential data: variables with positive associations, variables with negative associations, variables with no associations, and regression models.

For measurements of psychological or learning activities the instruments were also extracted.

### Study risk of bias assessment

The main author (J.F.C.) evaluated studies using the Medical Education Research Study Quality Instrument (MERSQI). A tool that evaluates 6 domains (study design, sampling, type of data, validity of the evaluation instrument, data analysis, and outcomes), it is composed of 10 items and the score could range from 5 to 18 [12].

### Synthesis methods

Data were tabulated in Microsoft Excel (Microsoft Corp., Redmond, WA, USA), then classified and analyzed to accomplish the main objective of this review. Each study was reviewed 3 times for a better classification of variables with positive associations, variables with negative associations, and variables with no association. The classification of data was conducted using the model of Alyahyan and Dustegor [11]; when in doubt about an instrument or variable, a senior researcher was approached. The extracted data were analyzed through a narrative synthesis, and then used to draw a figure to explain how all factors assessed interact with the ENAM score. Due to the heterogeneity and disparity among studies’ results, a meta-analysis was not performed.

### Reporting bias assessment

To minimize reporting bias, the gray literature and RENATI were searched, mainly because most of the ENAM research was conducted as graduate dissertations or theses, and these are considered to be peer-reviewed by the dissertation committee.

### Effect measures

Associated factors were reported as correlation coefficients (the Pearson correlation coefficient [r] or the Spearman rho [r_s_]), and the following criteria were used to assess the relationships as strong (r=0.7 to 1), moderate (r=0.4 to 0.6) or weak (r=0.1 to 0.3), both for negative and positive relationships. Regarding regression models, data were extracted as reported (odds ratio, prevalence ratio, or risk ratio) and, if it was adjusted, the factors used for adjustment were noted.

### Certainty assessment

Most of the studies were analytical and cross-sectional, with one case-control study. As established in the pyramid of evidence these studies are at the low end of the spectrum of evidence quality, but are those needed to answer the research question [13]. To assess the certainty of outcomes a P-value <0.05 with a confidence interval (CI) was the main criterion.

## Results

### Study selection

**Fig. 1.**
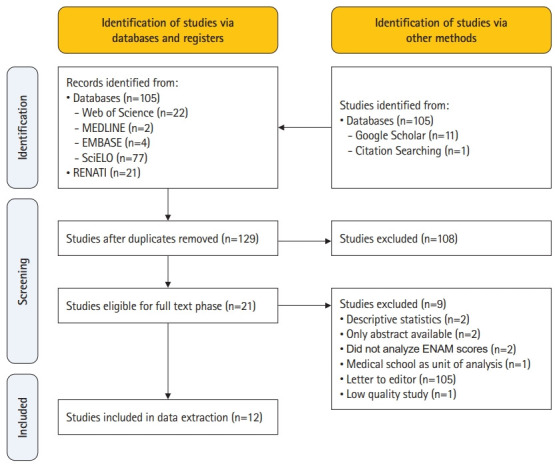
PRISMA (Preferred Reporting Items for Systematic Reviews and Meta-Analyses) flowchart of selected studies.

As shown in Fig. 1, 138 studies were identified using 4 electronic databases and one register, and 12 additional studies through the gray literature and citation searching. After duplicate removal, 129 studies were eligible for full-text review. Finally, 12 studies met the eligibility criteria for data extraction and analyses [7,14-24] (Table 1, Dataset 1).

### Study characteristics

Twelve identified studies included 38,500 students who took the ENAM. Most of the studies had an analytical cross-sectional design, with the exception of the study of Baldera Aquino and Alvarado Alva [15], which was a case-control study. The studies were published between 2011 to 2021, with most of them published between 2018 to 2021 (66.67%). Three studies were published in peer-reviewed scientific journals [7,14,18], while the remaining were published in RENATI as gray literature. The study samples ranged from 42 to 6,556 participants. The most evaluated factors were gender, GPA and age. The rate of disapproval of the ENAM ranged from 16% to 42.80%. The most frequently associated factors with the ENAM across studies were GPA, internship in EsSalud, older age (negative association) and regular academic status.

### Risk of bias in studies

The minimum score within 6 of the domains is 1, and the maximum score across all domains is 3. Accordingly, MERSQI scores range from 5 to 18. As stated in Table 2 [7,14-24], the mean MERSQI score was 10.33 (range, 9 to 12.5). Three studies enrolled 3 and more institutions [7,17,18]. The data collected were objective, with the exception of one study where the ENAM score was self-reported [24]. Due to the nature of the studies, all outcomes across studies were knowledge in the Kirkpatrick framework of the MERSQI.

### Synthesis of results

#### Factors associated with ENAM scores

Factors significantly associated with higher ENAM scores were GPA (n=8) [14,16-22], internship setting in EsSalud (n=4) [14,19,20,22], and regular academic status (n=3) [14,19,20]. Other factors positively associated were male gender (n=1), being single (n=1), higher socioeconomic level (n=1) and receiving career funding from one’s parents (n=1) [17]; among psychological factors, the use of metacognitive strategies, information seeking, and processing strategies (n=1) was positively associated [15]. Factors associated with lower scores on the ENAM were older age (being older than 25–26) (n=3) [17,18,22], being non-Peruvian (n=2) [7,17], and having moderate to high levels of test anxiety (n=1) [15] (Table 3).

#### Factors predicting ENAM scores

Although 7 studies conducted regression models [7,14-16,18,19,21], one did not conduct an adjusted model [15], one only assessed GPA and ENAM [18] and one did not state the criteria to conduct the regression model [19]; finally, the results of the 4 regression models included are shown in Table 4 [7,14,16,21].

### Reporting bias

Different studies were included, not only through peer-reviewed journals, but also the gray literature from dissertations.

### Certainty of evidence

There is a high level of certainty for GPA with ENAM, due to its association across studies and because learning theories support this finding. Regarding other factors, there is a low level of certainty although P-values were <0.05. This uncertainty is for the following reasons: (1) heterogeneity among studies, (2) observational designs, (3) absence of theory used to design and interpret the studies, and (4) differences in size across studies.

## Discussion

### Key results

**Fig. 2.**
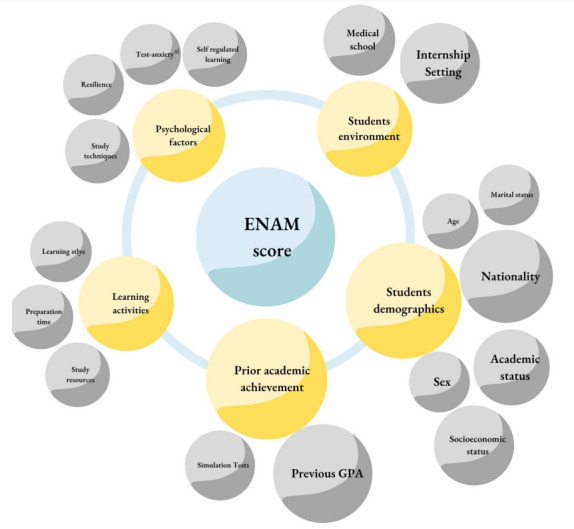
Factors associated with National Licensing Examination (ENAM) scores using an academic achievement model. ^a)^Test-anxiety refers to a type of anxiety that appears in an evaluative or testing setting (i.e., exam-related). GPA, grade point average.

This systematic review aimed to summarize the factors associated with ENAM scores among 12 studies. Although GPA remained the most studied factor, this review supports the fact that ENAM is a multifactorial outcome, as stated in previous models or reviews [11,25,26]. Therefore, in Fig. 2, a proposed model to understand how different factors interact with the ENAM score is presented.

### Interpretation

The results of the present systematic review showed that the ENAM score, as well as that of other licensing examinations, is not a one-factor product, but a multifactorial effect. Although GPA was the most studied factor and nationality was the strongest predictor, other factors such as age, marital status, academic status, gender, simulation tests, study resources, preparation time, learning styles, study techniques, resilience, test anxiety, self-regulated learning strategies, medical school, and internship setting play a role in the final score (Fig. 2).

### Comparison with previous studies

Previous studies conducted for the United States Medical Licensing Examination (USMLE) showed that previous academic achievement was correlated with higher USMLE scores [27]. A systematic review conducted in 2022 in the USMLE setting also found that step 1 scores, practice examinations, and GPA from high school, all indicators of previous academic achievement, were associated with higher step 2 scores [28]. Socioeconomic status plays an important role in education; as previously demonstrated by Jacobparayil et al. [28] and Giordano et al. [27], medical students with socioeconomic disadvantages and older age performed worst on USMLE examinations.

One finding that study resources do not have a major impact on ENAM scores contradicts the findings of a previous Best Evidence Medical Education review [29], according to which the use of an “off the shelf learning platform” was associated with higher scores on the USMLE, and associations were also found for the number of questions, reflection on mistakes, and rate of correct questions. This result differs from the findings of the present review, mainly because in the study of Sosa Espinoza [22], they only asked if students did or did not do practice questions.

Self-regulated learning was associated with higher ENAM scores, specifically the use of metacognitive, information seeking, and information processing strategies; these findings match those of Broadbent and Poon [30], according to whom self-regulated learning was associated with higher academic achievement. As a possible explanation for this finding, students with a higher use of metacognitive strategies can identify their mistakes on practice questions and adjust accordingly. Regarding test anxiety, the findings of this review are similar to those reported by Green et al. [31] in the USMLE setting, although in the same study test-taking strategies did not improve scores. However, a meta-analysis conducted on randomized controlled trials on higher education compared study skills training, behavior therapy, cognitive behavioral therapy, and combined therapies, showing that combined therapies had an estimated effect size of g=1.15 (95% CI, 0.33 to 1.96) on academic achievement [32].

### Limitations

One major limitation of this study is the higher rate of gray literature and heterogeneity in reports of desired outcomes, which made it unfeasible to conduct a meta-analysis. Another problem was the methodology and data collected among studies, in which few cofounders were analyzed.

### Implications

#### What does this mean for students?

For Peruvian medical students, this review will give them a locus of control on what they can improve in regard to ENAM scores: mainly self-regulated learning strategies, using high-utility study techniques, and focusing more on undergraduate medical education as it is the main predictor of ENAM scores. For my students, I would say, “Nothing beats a good undergraduate education.”

#### What does this mean for faculty, health policymakers, and managers?

For faculty, a bad ENAM score is not only a student’s problem, but a systematic problem, as shown in this review; it gives us opportunities to develop a curriculum to improve the ENAM score, some examples would be interventions on test anxiety, reducing the workload during the internship, and identifying low performers with progress tests or test simulations to use remediation programs.

#### What does this mean for future researchers?

For future researchers, I hope that this review gives an overview of what has been studied and which variables could be used to assess cofounders and interpret the results, and I hope that future researchers will design and conduct a high-quality randomized controlled trial.

#### What does this mean for the public?

I sincerely hope that the public understands that there is high inequity regarding factors associated with the ENAM, and it is not a one-way model in which medical students gain knowledge and regurgitate it, but as in every other aspect of life, social disparities have an impact and not everyone has the same background.

### Conclusion

A couple of years ago, one of my senior teachers told us that every medical student from Peru has the same opportunity to be a high achiever on the ENAM and it only depends on how “hard you study.”

By now, according to this review, I am glad to say that he was wrong, because the ENAM is a multifactorial phenomenon, and even if the GPA and nationality are the most important predictors, other factors are associated, such as academic achievement, student demographics, student environment, learning activities, and psychological factor, which had a role in the outcome. Therefore, the proposed model for the study of the ENAM score would help students, faculty, health policymakers, future researchers, and the public to better understand the ENAM.

## Figures and Tables

**Table 1.** Summary of characteristics of included studies

Study	Research type	Study design	Sample	Students’ demographic	Socioeconomic status	Students’ environment	Learning activities	Psychological factors	Prior academic achievement
Arenas-Significance [14] (2014)	Journal article	Cross-sectional	146	Sex; academic status	ND	Internship setting	ND	ND	GPA, simulation test
Baldera-Aquino [15] (2021)	Thesis	Case-control	123	Sex	Working status	Internship setting	ND	Self-regulated learning (CEVEPEAU); test anxiety (CAFEU)	ND
Flores Cohaila [16] (2020)	Thesis	Cross-sectional	45	Sex; age	ND	ND	ND	ND	GPA
Franco Miranda [17] (2020)	Thesis	Cross-sectional	187	Sex; age; marital status	Socioeconomic level; career funding	ND	Study resources	ND	ND
Huamaní [18] (2011)	Journal article	Cross-sectional	6,556	Nationality	ND	Medical school	ND	ND	GPA
Mendoza [7] (2021)	Journal article	Cross-sectional	30,750	Nationality	ND	Medical school	ND	ND	ND
Quispe [19] (2018)	Thesis	Cross-sectional	120	Sex; age; marital status; academic status	ND	ND	Study resources	ND	GPA
Ramos Supa [20](2020)	Thesis	Cross-sectional	72	Sex; age; academic Status	ND	Internship setting	Study resources, preparation time	Study technique, study habits (CASM 85), motivational scale (EME)	GPA, simulation test
Salazar Saavedra [21] (2015)	Thesis	Cross-sectional	256	Sex	ND	Internship setting	ND	ND	GPA, simulation test
Sosa Espinoza [22] (2018)	Thesis	Cross-sectional	98	Sex; age; academic status	Career funding	Internship setting	Study resources, preparation time	ND	GPA
Vojvodic Hernandez [23] (2019)	Thesis	Cross-sectional	42	Sex; age; marital status	ND	ND	ND	Resilience (CRE-U)	ND
Zuni Chavez [24] (2017)	Thesis	Cross-sectional	-	Sex; age; marital status	ND	Internship setting	Learning styles (Honey-Alonso Scale)	Self-perceived barriers	ND
						Medical school			

ND, not detected; GPA, grade point average; CEVEPEAU, Questionnaire for the Evaluation of Higher Education Student Learning Strategies; CAFEU, Questionnaire for Test Anxiety in Higher Education; EME, Motivational Education Scale; CRE-U, Resilience Questionnaire in Higher Education.

**Table 2.** Assessment of the quality of included articles with Medical Education Research Study Quality Instrument

Study	Study design	Sampling	Type of data	Validity of evaluation instrument	Data analysis	Outcome	Total score
Arenas-Significance [14] (2014)	1	0.5	3	0	3	1.5	9
Baldera Aquino [15] (2021)	2	0.5	3	2	3	1.5	12
Flores Cohaila [16] (2020)	1	0.5	3	0	3	1.5	9
Franco Miranda [17] (2020)	1	2	3	2	3	1.5	12.5
Huamaní [18] (2011)	1	2	3	0	3	1.5	10.5
Mendoza Chuctaya [7] (2021)	1	2	3	2	3	1.5	12.5
Quispe Chacon [19] (2018)	1	1	3	0	3	1.5	9.5
Ramos Supa [20] (2020)	1	0.5	3	2	3	1.5	11
Salazar Saavedra [21] (2015)	1	0.5	3	0	3	1.5	9
Sosa Espinoza [22] (2018)	1	0.5	3	0	3	1.5	9
Vojvodic Hernandez [23] (2019)	1	0.5	3	2	3	1.5	11
Zuni Chavez [24] (2017)	1	0.5	1	2	3	1.5	9

**Table 3.** Significant associations between variables and ENAM scores by domains

Domain	Factor	Significant association	No association
Students’ demographics	Age	Older age was associated with poorer outcomes across 3 studies [17,18,22].	In one study no association was found between age and ENAM score [16].
	Gender	Male gender was associated with better outcomes in ENAM score [17].	No association was found between gender and ENAM in 5 studies [14,16,19,20,22].
	Marital status	In one study being single was associated with higher ENAM scores [17].	-
	Nationality	In 2 studies, being non-Peruvian was associated with poorer scores in the ENAM [7,17].	-
	Academic status	Regular academic status was positively associated with the ENAM score [14,19,20].	In 2 studies, academic status was not associated with ENAM scores [16,21].
Socioeconomic status	Socioeconomic level	Higher economic level was associated with higher ENAM score [17].	-
	Career funding	Students whose careers were funded by their parents had higher ENAM scores than their peers [17].	There was no association between career funding and ENAM in the study of Sosa Espinoza and Sulca Correa [22].
	Working status	-	Baldera Aquino and Alvarado Alva [15] found that working status was not associated with ENAM scores.
Students’ environment	Medical school	Belonging to a public medical school or province medical school was associated with higher ENAM scores [7].	-
	Internship setting	Medical students who conducted their medical internships in EsSalud had better outcomes than their peers [14,19,20,22].	-
Learning activities	Study resources	Using study resources, mainly medical education videos through commercial learning platforms was positively associated with ENAM scores [17].	In 3 studies the use of commercial learning platforms was not associated with ENAM scores [19,20,22].
	Preparation time	Daily preparation time was associated with ENAM scores [22].	There was no association between preparation time in months and ENAM scores [20,22].
	Learning styles	Among learning styles, active learning was associated with ENAM scores [24].	-
Psychological factors	Self-regulated learning strategies	Metacognitive strategies, information seeking, and information processing strategies were positively associated [15].	Affective and resource management strategies were not associated with ENAM scores [15].
		A lack of motivation was negatively associated with ENAM scores [20].	
	Test anxiety	Moderate to high levels of test anxiety were negatively associated with ENAM scores [15].	-
	Resilience	All resilience domains were positively associated with ENAM scores [23].	-
Prior academic achievement	GPA	Higher GPA represented by a higher rank in class, or raw GPA was positively associated with ENAM scores [14,16-22].	-
	Progress test and simulation tests	Higher scores on practice tests (progress or simulation) were positively associated with ENAM scores in 2 studies.	In one study the scores on progress tests were not associated with ENAM scores [20].

ENAM, National Licensing Examination; EsSalud, Social Insurance in Peru; GPA, grade point average.

**Table 4.** Factors predicting the ENAM score among studies

Study	Previous academic achievement	Students’ demographics	Students’ environment	Outcome
Flores Cohaila [16] (2020)	GPA (>13): OR, 0.62 (95% CI, 0.01 to 0.7)	-	-	Disapproval of ENAM (<11); adjusted by gender, academic status, progress test, and GPA.
Mendoza Chuctaya [7] (2021)	-	Nationality: Cuba (PR, 8.45; 95% CI, 7.93 to 8.99); Venezuela (PR, 2.26; 95% CI, 1.93 to 2.65); Bolivia (PR, 1.66; 95% CI, 1.61 to 1.71)	Private medical school: PR, 1.42 (95% CI, 1.37 to 1.47)	Disapproval of ENAM (<11); adjusted by year that students took the ENAM, class GPA, nationality, and medical school.
Arenas-Significance [14] (2021)	Academic honors: OR, 0.24 (95% CI, 0.1 to 0.58)	Regular academic status: OR, 0.36 (95% CI, 0.14 to 0.88)	-	Disapproval of ENAM (<11); adjusted by academic status, internship setting, and academic honors
Salazar Saavedra [21] (2015)	GPA^a)^: OR, 10.94 (95% CI, 4.12 to 28.98)	Male gender: OR, 2.733 (95% CI, 1.30 to 5.74)	Internship in EsSalud: OR, 6.419 (95% CI, 2.07 to 19.87)	ENAM score >12.5; adjusted by gender, internship setting, academic honors, GPA, number of simulation tests, and graduation modality.

ENAM, National Licensing Examination; GPA, grade point average; OR, odds ratio; CI, confidence interval; PR, prevalence ratio;

a) For each point in GPA, the OR increased by 10.94. Academic honors: being on equal or higher than the 66th percentile. Graduation modality: If the acquisition of the degree was by thesis dissertation or by an examination.

## Footnotes

**Data availability**
Data files are available from Harvard Dataverse: https://doi.org/10.7910/DVN/69GIM5 Dataset 1. Raw analysis data of 12 selected articles

## Supplementary materials

Supplementary files are available from Harvard Dataverse: https://doi.org/10.7910/DVN/69GIM5

Supplement 1. Search strategy and the data source.

Supplement 2. Audio recording of the abstract.
