# Supplementary material for: Factors associated with medical students’ scores on the National Licensing Exam in Peru: a systematic review
Source: J Educ Eval Health Prof. 2022 Dec 29;19:38. doi: 10.3352/jeehp.2022.19.38 (PMC9889888; doi:10.3352/jeehp.2022.19.38)
Supplement: Supplementary file 2 — Supplement 1. Search strategy and the data source. [file jeehp-19-38-suppl.docx]

**Supplement 1.** Search strategy and the data source

**Table 1.** Search strategy

|  | Category | Term | Web of Science | MEDLINE | EMBASE | Scielo | RENATI |
| --- | --- | --- | --- | --- | --- | --- | --- |
| #1 | ENAM | (“ENAM”) OR (“Examen Nacional de Medicina”) OR (“Licensing examination”) |  |  |  |  |  |
| #2 | Associated Factors | (“Correlation”) OR (“Concordance”) OR (“Differences”) OR (“Association”) OR (“Associated Factors”) |  |  |  |  |  |
| #3 | Peru | (“Peru”) OR (“Peruvian”) |  |  |  |  |  |
| #4 | Combined | #1 AND #2 | 22 | 2 | 4 | 77 | 21 |

**Table #2.** Details search terms in databases

|  | Databases | Search strategy |
| --- | --- | --- |
| #1 | MEDLINE | ((“ENAM”) OR (“Examen Nacional de Medicina”) OR (“Licensing examination”)) AND ((“Correlation”) OR (“Concordance”) OR (“Differences”) OR (“Association”) OR (“Associated Factors”)) AND ((“Peru”) OR (“Peruvian”)) |
| #2 | EMBASE | ((“ENAM”) OR (“Examen Nacional de Medicina”) OR (“Licensing examination”)) AND ((“Correlation”) OR (“Concordance”) OR (“Differences”) OR (“Association”) OR (“Associated Factors”)) AND ((“Peru”) OR (“Peruvian”)) |
| #3 | Scielo | ((“ENAM”) OR (“Examen Nacional de Medicina”) |
| #4 | Web of Science | ((“ENAM”) OR (“Examen Nacional de Medicina”) |
| #5 | RENATI | ((“ENAM”) OR (“Examen Nacional de Medicina”) |
